# Individual differences in eating attitudes are associated with distinct resting-state functional connectivity patterns in healthy adults

**DOI:** 10.1007/s11682-026-01212-z

**Published:** 2026-11-11

**Authors:** Bianca Borsarini, Stefania Valentini Konstantopoulou, Emilie Marti, Sélim Coll, Radek Ptak

**Affiliations:** 1 Network, Plasticity and Modulation (NetPM) Laboratory, Department of Clinical Neurosciences, Faculty of Medicine, University of Geneva, Geneva, Switzerland; 2 Division of Neurorehabilitation, Department of Clinical Neurosciences, Geneva University Hospitals, Geneva, Switzerland

**Keywords:** Resting-state, Functional Connectivity, Vulnerability, Eating disorders, Obesity

## Abstract

Eating disorders (EDs) and obesity are multifaceted conditions that reflect extremes of over- and under-nutrition and may cause significant life-altering complications. The individual susceptibility to potential development of EDs or obesity is determined by specific eating behaviors and attitudes toward food. Whether this behavioural vulnerability is also associated with specific neural connectivity patterns is unknown. This study aimed to investigate associations between resting-state functional connectivity (rs-FC) and nonpathological premorbid attitudes toward food in healthy adults. Forty-eight healthy adults (mean age = 61.79 ± 11.07) underwent structural and functional MRI acquisitions on a 3-T MRI scanner. Attitudes toward food and eating were assessed using the Adult Eating Behavior Questionnaire (AEBQ). The relationship between seed-based connectivity and appetitive traits derived from the AEBQ was examined with general linear model group-level inferences, focusing on brain regions involved in behavioural control and salience processing. *Overeating susceptibility* was associated with stronger rs-FC between the right inferior frontal gyrus and the posterior cingulate/precuneus, as well as greater coupling between limbic/peri-limbic areas and reward–salience as well as prefrontal-control regions. In contrast, *undereating vulnerability* was associated with enhanced connectivity between right hippocampus and left opercular–insular regions. Finally, *food fussiness* was associated with the coupling between bilateral superior-frontal and prefrontal regions. Our findings suggest that *increased* rs-FC, specifically between regions involved in executive control, decision making, salience, reward evaluation and self-referential processing, predict attitudes toward food and eating that may increase individual susceptibility to EDs and obesity in healthy adults.

**Supplementary Information:**

The online version contains supplementary material available at https://doi.org/10.1007/s11682-026-01212-z.

## Introduction

Eating disorders (EDs) and obesity are debilitating illnesses of heterogeneous aetiology, marked primarily by altered eating attitudes that eventually may cause significant life-threatening complications (American Psychiatric Association, 2013; World Health Organization, 2024). Whereas EDs are full-fledged *psychiatric* illnesses associated with one of the highest rates of morbidity and mortality amongst all mental disorders (Arcelus et al., 2011; Smink et al., 2012), obesity is primarily a *physical* condition in which an excessive accumulation of body fat determines a latent, but active state of persistent health risk, linked to severe underlying metabolic, inflammatory and hormonal disruptions (World Health Organization, 2024). The aetiology of EDs and obesity is highly heterogeneous: genetic, biological, psychological, developmental and socio-cultural factors participate to their emergence (Bulik et al., 2022; Hawton et al., 2025). Both disorders are also conceived as neurobiologically rooted (Carnell et al., 2012; Frank, 2015; Frank et al., 2019; Ju et al., 2025) since characterized by functional and structural abnormalities in the brain (Bulik et al., 2022; Tanaka et al., 2020​). The complex pathogenesis of EDs and obesity renders the management of both conditions challenging and often poorly effective (Grilo, 2024; Karasu, 2012). Additionally, diagnosis is often delayed (Kalindjian et al., 2022; Lydecker & Grilo, 2017) and the instatement of preventive procedures is demanding (Le et al., 2017).

Factors predisposing for EDs and obesity are of various nature (from genetic to sociocultural) and generally become manifest within a specific age range (from infancy to adulthood) (Barakat et al., 2023; Carnell et al., 2023). Pathological attitudes such as morbid eating and dietary habits, nutritional intake and food choices, play a major role for the emergence and long-term maintenance of EDs and obesity (American Psychiatric Association, 2013; World Health Organization, 2024; Derks et al., 2024). Eating behavior is determined by interactions between genes and environment (De Castro, 2010): social and cultural environments moderate the expression of genetically-determined appetitive tendencies, whether restrictive or excessive (Carnell et al., 2023; De Castro, 2010; Jansen et al., 2023). This suggests that individual appetitive traits emerge early in development (Carnell et al., 2008; Jansen et al., 2023; Derks et al., 2024), exert long-term influences on eating behavior and body weight regulation, and have identifiable biological generators (Derks et al., 2024; Jansen et al., 2023; Konttinen et al., 2015; Wardle & Carnell, 2009).

Appetitive traits are conceptualized as persistent predispositions toward food and are commonly categorized as reflecting food *approach* or food *avoidance* (Carnell & Wardle, 2008; Jansen et al., 2023). In the context of EDs or obesity such predispositions either represent behavioral risk factors determining the individual susceptibility to potential development of disease, or their early neurobehavioral markers (Derks et al., 2024; Konttinen et al., 2015). In the light of prevention efforts and tailored interventions, an early and precise identification of premorbid appetite traits in individuals at risk is of critical importance (Grange & Loeb, 2007; Koreshe et al., 2023).

Here, we posit that appetitive traits are linked to specific trait-like neural signatures (Carnell & Wardle, 2008; Konttinen et al., 2015; Wardle & Carnell, 2009), which should be reflected in specific patterns of neural networks. Resting-state functional connectivity (rs-FC) refers to the temporal correlations of spontaneous signal fluctuations between different brain regions when a person is not engaged in a task. Rs-FC measured with functional MRI enables mapping the organization of intrinsic brain networks and their alterations in health and disease (Biswal & Uddin, 2025). Recent MRI-studies on clinical samples diagnosed with ED or obesity have found alterations of rs-FC networks specifically involved in reward and salience evaluation, self-referential processing, decision-making and cognitive control (Chen et al., 2024; Donofry, Stillman et al., 2020b). At the same time, rs-FC investigations in non-clinical samples targeting non-pathological appetitive traits are scarce. In preschool children, Chodkowski and colleagues observed first a positive association between adiposity and rs-FC in brain regions involved in impulsive behaviors, and then that both adiposity and impulsivity were, respectively, positively associated with food approach behaviors and negatively associated with food avoidance traits (Chodkowski et al., 2016). Chen and colleagues observed in young adults (solely 17–25 years of age) a negative association between the severity of overt ED-symptoms and rs-FC in several brain networks (Chen et al., 2021). However, so far, no study has investigated whether *non-pathological* appetitive traits reflecting approach or avoidance attitudes are associated with specific rs-FC patterns in the general *adult* population.

Against this background, the present study aimed to investigate potential associations between fMRI rs-FC and attitudes towards food in the general population. We hypothesized that greater food-approach tendencies and the susceptibility to overeating are accompanied by increased rs-FC in brain networks involved in salience detection and reward processing, while food-avoidance and susceptibility to dietary restriction are associated with enhanced rs-FC in networks engaged in executive control, decision making and interoceptive awareness.

## Methods and materials

### Sample

The recruitment was performed through online advertisement, flyers distributed in communal areas at the University Hospitals of Geneva, the University of Geneva and the Foundation Campus Biotech Geneva (Switzerland), and word-of-mouth referrals of participants’ friends and relatives. Interested subjects were contacted telephonically to check inclusion criteria, to obtain consent and to schedule the experimental study session. Participants of both sexes were enrolled if they matched the following criteria: being between 30 and 80 years old, in good current health (no diagnosed psychiatric or medical condition susceptible to interfere with eating behavior) and eligibility for an MRI examination.

### Measures

#### Socio-demographic and anthropometric data

Sociodemographic information and anthropometric data were collected for each participant during the enrolment, including contact information, date of birth, native language, biological sex, ethnicity, educational level (years of schooling, coded as follows: Level 1= < 11 years; Level 2= 11–14 years; Level 3= > 14 years), handedness, height and weight.

#### Experimental procedure

Participants concluded a single experimental session at the Foundation Campus Biotech Geneva. The session involved an MRI acquisition of 30 min total duration, followed by three questionnaires: the Fatigue Severity Scale (FSS) (Krupp et al., 1989), the Hospital Anxiety and Depression Scale (HADS) (Zigmond & Snaith, 1983) and the Adult Eating Behavior Questionnaire (AEBQ) (Hunot et al., 2016). Details about HADS and FSS, together with related descriptives, frequency graphs and exploratory correlations can be found in Supplementary Materials, Section 1SM.

Imaging acquisition was performed using a Siemens MAGNETOM Prisma 3 T MRI scanner, equipped with a 64-channel head coil. The acquisition protocol included the following sequences:

1. A high-resolution, structural T1-weighted mp2rage sequence (isotropic voxel resolution = 1.0 mm^3^, repetition time (TR) = 2300 ms, echo time (TE) = 1.96 ms, slice thickness = 1.0 mm, 3D distortion correction applied);
2. A gradient-echo echo-planar imaging (EPI) sequence with multiband acceleration (TR = 720 ms, TE = 30 ms, flip angle = 50°, 56 slices, voxel size = 2.0 × 2.0 × 2.5 mm³). A total of 440 volumes were collected over 5 min and 26 s, while participants were instructed to rest with eyes closed and not to fall asleep;
3. A dual gradient-echo fieldmap sequence to enable correction of susceptibility-induced geometric distortions (TR = 550 ms; TE₁ = 5.19 ms, TE₂ = 7.65 ms; ΔTE = 2.46 ms; flip angle = 60°; slices = 56; slice thickness = 2.5 mm).

All images were reviewed in real-time for quality assurance, including checks for motion artifacts and anatomical abnormalities.

#### Eating attitudes assessment

Appetitive traits were assessed using the Adult Eating Behavior Questionnaire (AEBQ), a 35-items, self-report psychometric tool for non-clinical adult populations (> 13 years) to measure eight specific premorbid aspects of eating behavior (Hunot et al., 2016). These eight eating subscales are clustered into two overarching dimensions:

1. *Food Approach Scales*: including the four subscales Enjoyment of Food (EF – 3 questions), Emotional Overeating (EOE – 5 questions), Food Responsiveness (FR – 4 questions) and Hunger (H – 5 questions) (Hunot et al., 2016);
2. *Food Avoidance Scales*: comprising the other four subscales Emotional Undereating (EUE – 5 questions), Food Fussiness (FF – 5 questions), Slowness in Eating (SE – 4 questions) and Satiety Responsiveness (SR – 4 questions) (Hunot et al., 2016).

The response options for each item were ‘strongly disagree’ (1), ‘disagree’ (2), ‘neither agree not disagree’ (3), ‘agree’ (4) and ‘strongly agree’ (5). For every subscale, an average score was calculated out of the relevant items.

## Data analyses

All statistical analyses about non-imaging data were carried out using JASP Version 0.95.3 (JASP Team, 2025). Statistical significance was set at *p* <.05.

### Eating behavior covariates

We first normalized all AEBQ scores and then computed a principal component analysis (PCA) separately for each of the two theoretical AEBQ domains (approach vs. avoidance) to reduce the number of variables. This method added data-driven weighting while ensuring theory-driven structure: scores were not affected by conflicting cross-domain variance, since food approach and food avoidance traits belonged to opposite constructs. Components were accepted if justified by eigenvalues > 1 to best reflect *all* the latent dimensions within that theoretical domain. This approach identified the following components:

1. *Overeating Susceptibility*: derived from the subscales Emotional Overeating (EO), Hunger (H) and Food Responsiveness (FR).
2. *Food Enjoyment*: derived from the subscale Enjoyment of Food (EF).
3. *Food Fussiness*: derived from the subscales Food Fussiness (FF) and Satiety Responsiveness (SR).
4. *Undereating Susceptibility*: derived from the subscales Emotional Undereating (EU) and Slowness in Eating (SE).

All the PCA specifics, including components loadings and path graphs, are reported in Supplementary Materials, Section 2SM.

### Resting-state fMRI data analysis

Analyses of fMRI data were performed using CONN (Nieto-Castanon & Whitfield-Gabrieli, 2022; Whitfield-Gabrieli & Nieto-Castanon, 2012) and SPM25 (Penny et al., 2011).

#### Preprocessing and denoising

Functional and anatomical data were preprocessed using a modular preprocessing pipeline, denoised using a standard denoising pipeline (Nieto-Castanon, 2020a, b), followed by bandpass frequency filtering between 0.008 Hz and 0.09 Hz.

#### First-level analysis

Seed-based connectivity maps (SBC) and ROI-to-ROI connectivity matrices (RRC) were estimated characterizing the patterns of functional connectivity based on 164 cortical ROIs of known HPC-ICA networks (Nieto-Castanon 2020c) and the Harvard-Oxford atlas (Desikan et al., 2006). Functional connectivity strength was represented by Fisher-transformed bivariate correlation coefficients from a weighted general linear model (weighted-GLM), defined separately for each pair of seed and target areas, modelling the association between their BOLD signal time series (Nieto-Castanon 2020c).

#### Second-level analysis

Group-level analyses were performed using a GLM approach (Nieto-Castanon 2020c). For each individual voxel, a separate GLM was estimated: the four weighted PCA-derived eating behavior dimensions, entered as second-level covariates, were the regressors. Biological age was used as a covariate to adjust for possible age effects. Seed-to-voxel analyses were performed considering specific anatomical regions of interest (ROIs) as seeds, pre-selected based on their involvement in hunger/satiety responses (insula, hippocampus), reward processing (nucleus accumbens, amygdala), regulation of food intake and eating behavior (striatum, cingulate cortex, superior, middle and inferior frontal gyrus) (Chae et al., 2023). Inferences were performed at the level of individual clusters (groups of contiguous voxels). Results were thresholded using a combination of a cluster-forming *p* <.001 voxel-level threshold, and a familywise corrected p-FWE < 0.05 cluster-size threshold.

## Results

Fifty participants completed all study sections. Two participants were excluded after preprocessing and denoising fMRI data due to excessive motion artifacts. The final sample consisted of 48 participants (28 females). Sex subgroups within our sample did not differ for age (t = 0.932, *p* =.356), body mass index (BMI) (t = −1.103, *p* =.276), education (χ^2^ = 1.660, *p* =.436), depression scores (*U* = 262, *p* =.708), anxiety scores (*U* = 293.5, *p* =.784) nor fatigue (*U* = 250.5, *p* =.542) (Table 1).

**Table 1:** Demographic, anthropometric and psychological characteristics of the sample

Variable		Descriptives
**Age (years)**, Mean (SD)		61.79 (11.07)
**BMI (kg/m**^**2**^**)**, Mean (SD)		23.95 (2.78)
**Sex**, n (%)	F	28 (58.33%)
	M	20 (41.67%)
**Education**, n (%)	Level 1 (< 11 years)	2 (4.17%)
	Level 2 (1–14 years)	8 (16.66%)
	Level 3 (> 14 years)	38 (79.17%)
**HADS Depression**, Median (Range)		2 (0–12)
**HADS Anxiety**, Median (Range)		6 (0–16)
**FSS Fatigue**, Median (Range)		2.05 (1.00–4.78)

For what concerns the affective and fatigue-related characteristics of the sample, out of 48 individuals only 2 subjects scored for moderate depressive symptoms, 4 for moderate anxiety and 3 for mild fatigue (Supplementary Materials, Section 1SM). Non-parametric Spearman’s correlations have been run for exploratory purposes between HADS, FSS scores and AEBQ-derived eating attitudes. Significant *positive* correlations have been found, respectively, between overeating susceptibility, depression scores (ρ = 0.503, *p* <.001), anxiety scores (ρ = 0.314, *p* =.030) and fatigue (ρ = 0.355, *p* =.013); between respectively food fussiness, depression scores (ρ = 0.376, *p* =.008) and anxiety scores (ρ = 0.367, *p* =.010). A significant *negative* correlation has been observed between food enjoyment and anxiety scores (ρ = −0.358, *p* =.013) (Supplementary Materials, Section 1SM).

### Eating attitudes and resting-state functional connectivity

The descriptive statistics concerning the scores for each AEBQ dimension, subscale and PCA-derived eating attitude are reported in Table 2 for the whole sample. Sex subgroups did not differ statistically for any of the AEBQ dimension or subscale. AEBQ scores’ full descriptives with sex and educational level stratification are reported in Supplementary Materials, Section 3SM.

**Table 2:** AEBQ scores descriptives for the whole sample. Responses ranged from a minimum of 1 to a maximum of 5. For each subject, an average score was calculated for every dimension, subscale and PCA-derived eating attitude

						Descriptives	
**AEBQ Variables**		**Median**	**Mean**	**SD**	**Range**	**Min**	**Max**
**Dimensions**	Food Approach	2.850	2.891	0.540	2.313	1.863	4.175
	Food Avoidance	2.394	2.380	0.472	1.825	1.438	3.263
**PCA-derived** **Eating Attitudes**	**Subscales**	**Median**	**Mean**	**SD**	**Range**	**Min**	**Max**
**Overeating Susceptibility**	Food Responsiveness	2.750	2.884	0.649	2.750	1.750	4.500
	Emotional Overeating	2.000	2.175	0.874	3.200	1.000	4.200
	Hunger	2.400	2.416	0.829	3.400	1.000	4.400
**Food Enjoyment**	Enjoyment of Food	4.300	4.088	0.648	3.000	2.000	5.000
**Food Fussiness**	Food Fussiness	1.800	1.729	0.697	4.000	1.000	5.000
	Satiety Responsiveness	2.450	2.404	0.639	2.750	1.250	4.000
**Undereating Susceptibility**	Slowness Eating	2.550	2.584	0.863	3.500	1.000	4.500
	Emotional Undereating	2.800	2.804	0.902	3.400	1.000	4.400

Group-level seed-to-voxel analyses revealed significant associations between individual differences in premorbid, eating-behavior traits and resting-state functional connectivity. Summary statistics for all significant clusters are reported in Table 3. Spatial coordinates for clusters and ROIs refer to the Montreal Neurological Institute MNI-ICBM 152 template (Mazziotta et al., 2001; J. C. Mazziotta et al., 1995).

**Table 3:** Significant clusters for seed-to-voxel resting-state connectivity analyses testing associations with eating-behavior covariates

Eating Behavior Covariate	Seed	Cluster peak (x, y, z)	Cluster size (voxels)	FWE *p*	Anatomical label
**Overeating** **Susceptibility**	**Right Inferior Frontal Gyrus (rIFG)**	+ 6, − 54, + 16	660	< 0.001	Retrosplenial Posterior Cingulate cortex, precuneus
	**Cingulate Cortex (CC)**	–32, + 18, − 4	271	0.001	Left anterior insula, left OFC, ACC/insula boundary
	**Left Hippocampus**	+ 34, + 2, − 8	204	0.005	Right putamen, right amygdala, right insula, OFC (Limbic-subcortical network)
		+ 56, + 28, +16	164	0.017	Right IFG and middle FG (fronto-temporal extension)
	**Right** **Amygdala**	+ 50, + 18, +28	140	0.032	Right IFG and right middle FG (fronto-temporal extension)
		–50, + 12, +32	126	0.049	Left IFG and left middle FG extending into precentral gyrus
**Food** **Fussiness**	**Right Superior Frontal Gyrus (rSFG)**	–22, − 104, − 6	161	0.021	Left posterior occipital cortex (left visual pole)
	**Right Inferior Frontal Gyrus (rIFG)**	–2, + 12, +60	186	0.010	Bilateral Superior FG, left Juxtapositional Lobule/SMA
		–54, + 40, − 6	152	0.025	Left IFG, left OFC
**Undereating** **Susceptibility**	**Right Hippocampus**	–40, + 6, +6	153	0.025	Left frontal operculum, central operculum, left anterior insula, and IFG

#### Overeating susceptibility

**Fig. 1:**
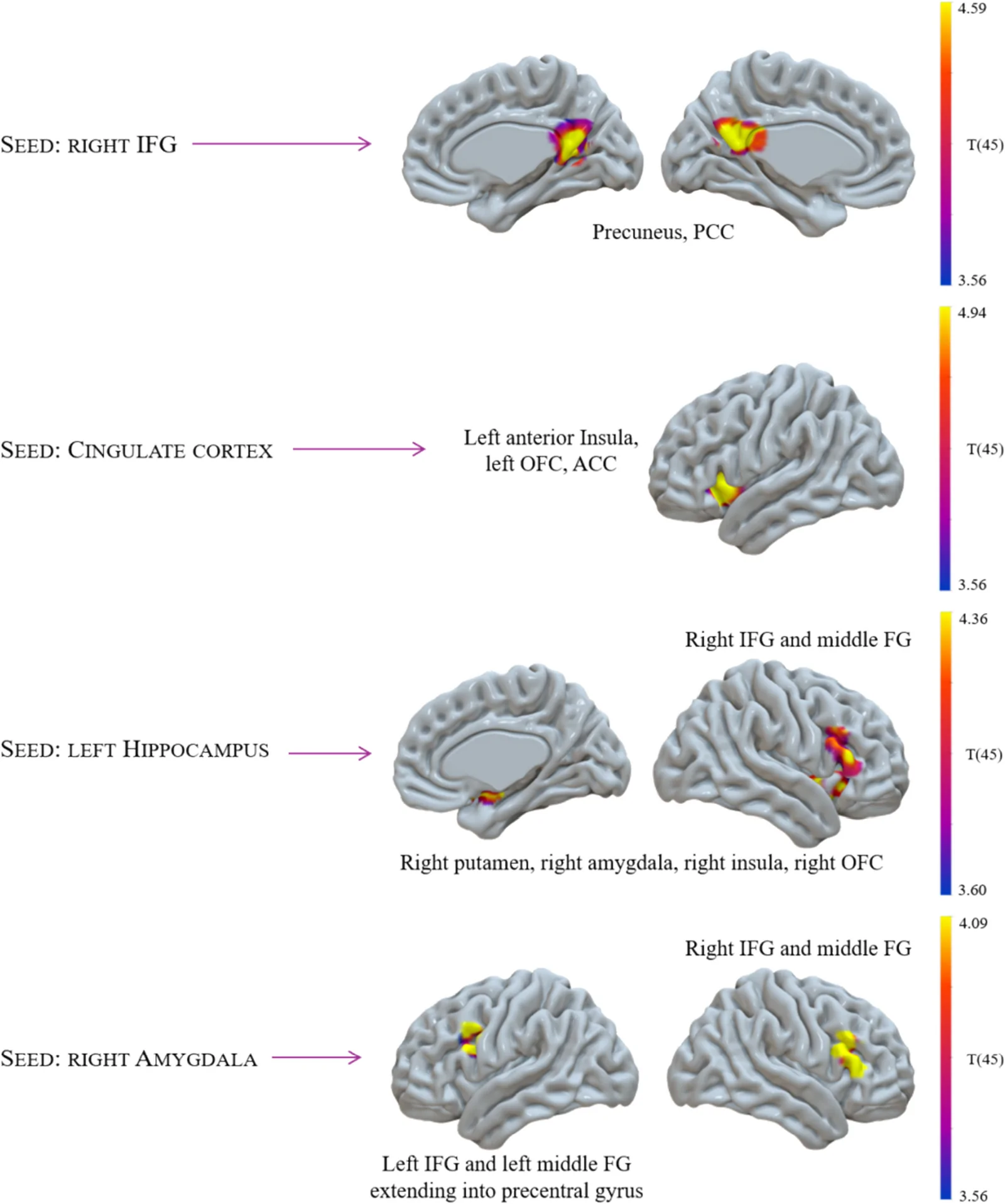
Results of global seed-based resting-state connectivity analyses associated with overeating susceptibility. Reported significant clusters indicate increased functional connectivity with the seeds

Overeating susceptibility was positively associated with functional connectivity between the right inferior frontal gyrus (IFG) and a large medial parietal cluster, primarily involving the retrosplenial posterior cingulate cortex and partially the precuneus. Overeating was similarly associated with FC between the cingulate cortex, the left anterior insula and left frontal orbital cortex (OFC). We found a third positive association between the left hippocampus and the right putamen, amygdala, insula, as well as the right inferior and middle frontal gyrus. Finally, overeating susceptibility was positively associated with greater coupling between the right amygdala and lateral frontal cortex in both hemispheres. Overall, alterations among prefrontal control hubs, limbic/subcortical regions and default-mode, reward-salience and valuation circuits might accompany susceptibility to overeating (Fig. 1).

#### Food fussiness

**Fig. 2:**
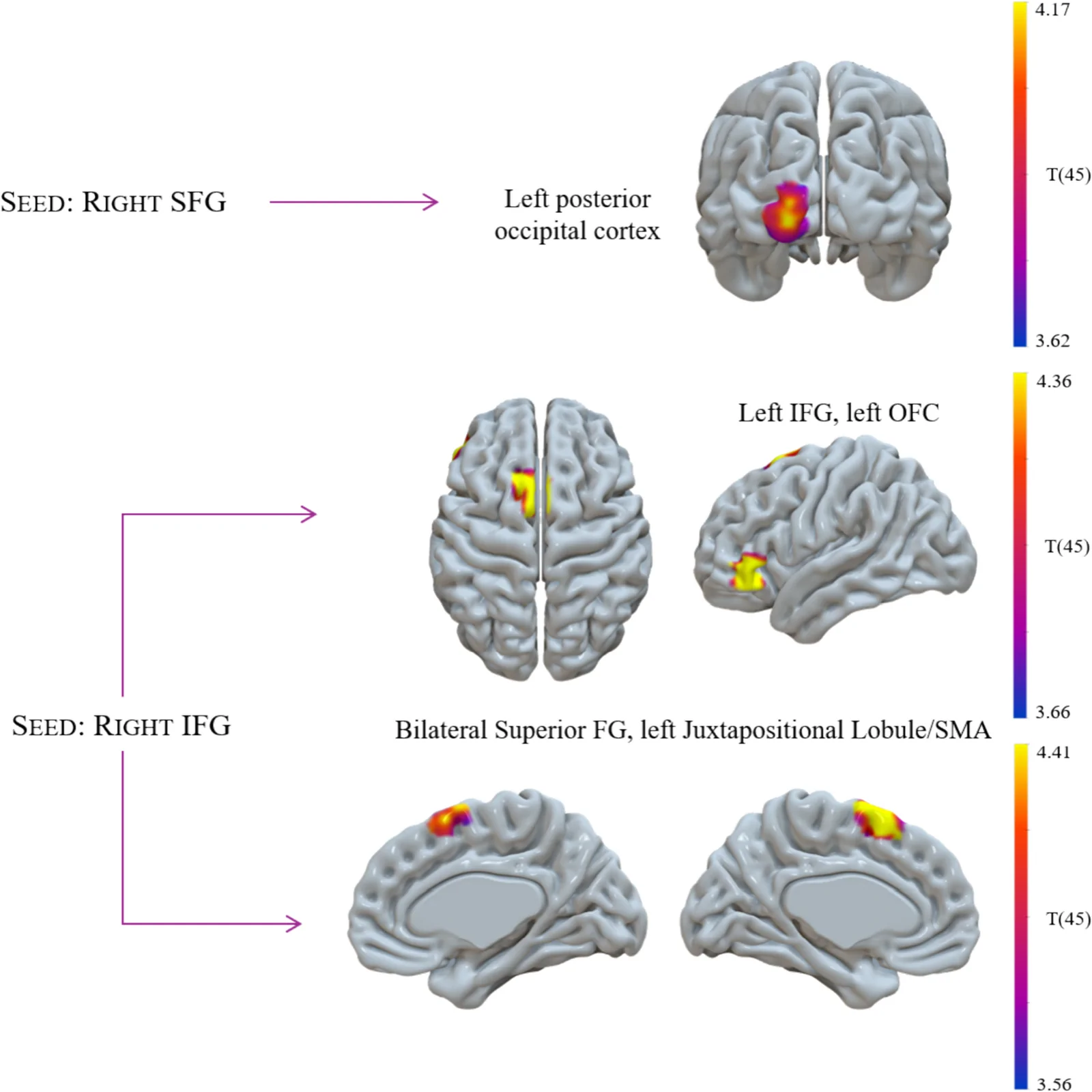
Results of global seed-based resting-state connectivity analyses associated with food fussiness. Reported significant clusters indicate increased functional connectivity with the seeds

Food fussiness scores were positively related to connectivity between the right superior frontal gyrus (SFG) and left posterior occipital cortex. There was also an association between the right IFG and bilateral supplementary motor area (SMA) on the one hand, and the left IFG (pars triangularis) on the other hand. Higher levels of food fussiness were thus associated with increased resting-state connectivity in networks involved in executive control, self-referential processing, and decision-making (Fig. 2).

#### Undereating susceptibility

**Fig. 3:**
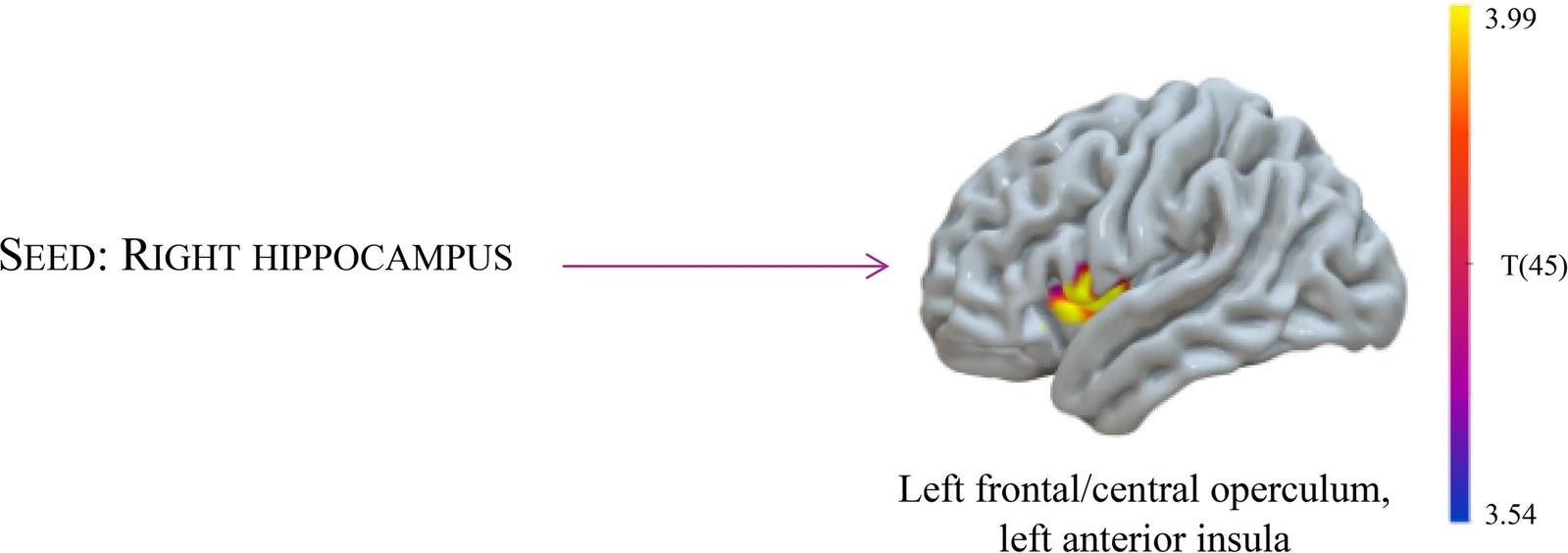
Results of global seed-based resting-state connectivity analyses associated with undereating susceptibility. Reported significant cluster indicates increased functional connectivity with the seed

Undereating susceptibility was positively associated with connectivity between the right hippocampus and the left frontal operculum and anterior insula (Fig. 3).

#### Food enjoyment

We found no significant associations between food enjoyment and functional connectivity.

## Discussion

We observed distinct associations between eating attitudes and functional connectivity patterns between regions involved in large-scale networks related to saliency and reward processing, behavioral control and interoceptive awareness. Importantly, these neural patterns emerged in a non-clinical sample, supporting the notion that vulnerability to maladaptive eating behaviors may be reflected in stable, trait-like functional brain connectivity irrespective of the actual presence of clinically relevant symptoms.

Overeating susceptibility was associated with alterations in rs-FC involving prefrontal regions relevant for top-down cognitive control, default mode network (DMN) hubs, and limbic-subcortical circuits. These findings align with the theory of food addiction (Schulte et al., 2021), which conceives overeating and binge-eating as a result of malfunctioning top-down prefrontal control coupled with hyperresponsivity of hedonic/reward systems, overriding homeostatic regulation of food intake (Smith & Robbins, 2013; Wijngaarden et al., 2015; Ravichandran et al., 2021). The strongest predictor of overeating was connectivity between the right inferior frontal gyrus (rIFG) and the retrosplenial posterior cingulate cortex (PCC). These regions are central DMN nodes, which is implicated in self-referential processing, internally oriented cognition, and value-driven decision making (Buckner & DiNicola, 2019; Yeshurun et al., 2021). Increased coupling between right IFG and PCC may here reflect enhanced interchange between self-referential cognitive control and internally guided evaluation processes (Donofry et al., 2020a, b). Previous findings on binge-eating or clinical obesity (Chen et al. 2024; Donofry et al., 2020a, b; Stopyra et al. 2019) highlighted an association between DMN-PFC hyperconnectivity and increased food cravings, excessive self-referred rumination and bodily awareness, and amplified anticipated estimation of rewards. Thus, DMN-PFC connectivity could reflect enhanced processing of food cues’ leading to overestimated food salience going beyond the mere experience of reward or pleasure (Simon et al. 2016; Donofry et al., 2020a, b). Overeating susceptibility was also associated with increased connectivity between the cingulate cortex and the anterior insula, the orbitofrontal cortex (OFC) and the anterior cingulate cortex. These regions are key hubs of the Salience Network (SN), responsible for identifying homeostatically-relevant stimuli by rating external variables and internal needs (Seeley, 2019), allocating attentional resources to them (Parr & Friston, 2017) and switching between self-oriented focus and externally oriented attention (Seeley, 2019; Schimmelpfennig et al., 2023). Within SN, the insula bridges prefrontal executive control with proprioception, internal states and self-awareness (Parr & Friston, 2017; Schimmelpfennig et al., 2023). Greater cingulate–insula–OFC coupling may reflect heightened awareness of internal bodily states, possibly leading to compromised regulatory control of food intake (Kullmann et al., 2013; Kessler et al., 2016; Wang et al., 2022). Overeating susceptibility was further linked to higher connectivity between the left hippocampus and brain regions processing reward, including IFG/MFG (Han et al., 2018), putamen, amygdala, insula, and OFC (Seeley, 2019). The hippocampus plays a core role in regulating human feeding behavior based on meal-related memories (Kanoski & Grill, 2017; Stevenson & Francis, 2017) by integrating contextual food-related experiences with affective salience, learned reward associations, external eco-social factors, mnemonic information related to food availability and peri-ingestive signals like hunger and satiety (G.-J. Wang et al., 2006). This enables hippocampal neurons to adapt feeding choices based on earlier integration between external contextual cues with internal homeostatic status (Kanoski & Grill, 2017). Altered connectivity between hippocampus, the reward system and the salience network has been repeatedly reported in individuals with obesity, clinical overeating or impaired appetite regulation (Alonso-Alonso et al., 2015; Kanoski & Grill, 2017). The observed stronger hippocampal–striatal–limbic coupling may promote the retrieval of rewarding food memories and their related emotional salience (Kanoski & Grill, 2017), while enhanced hippocampal-frontal connectivity might bias food-related decision making modulating eating behavior towards prolonged excessive intake (Stevenson and Francis 2017; Donofry et al., 2020a, b). Greater overeating susceptibility was finally associated with increased rs-FC between the right amygdala and the medial-inferior frontotemporal regions, bilaterally. The amygdala is a subcortical SN component critically involved in emotional learning, significance-based and reward-driven eating, particularly under conditions of distress or negative affectivity (Petrovich & Gallagher, 2003; Izadi & Radahmadi, 2022). Abnormalities related to the amygdala, prefrontal regions and their interconnectivity have been previously observed in association to emotional eating, impulsivity, altered decision-making and reduced inhibitory control over food intake (García-García et al., 2014), both in young (Chodkowski et al., 2016; Kim et al., 2020) and adult populations (Ravichandran et al., 2021). Within this scenario, also the significant positive correlations observed in our sample between overeating tendencies and emotional factors, such as non-clinical depression and anxiety traits, find a coherent contextualization: indeed, both depression and anxiety have been observed to be significantly correlated to emotional overeating in the general adult population (Dakanalis et al., 2023; Ljubičić et al., 2023), and to be accompanied by greater rs-FC in homologous hubs belonging to DMN (Greicius et al., 2007) and Salience Network (Pannekoek et al., 2013). Altogether, our findings indicate that overeating vulnerability is characterized by enhanced integration between salience, reward, control, memory, and self-referential networks, possibly increasing sensitivity and responses to food-related cues, even in the absence of hunger and in individuals without a pathological relationship with food. Positive correlations between overeating susceptibility and negative affectivity further support the proposed speculation.

Contrasting with overeating, in our sample food fussiness was associated with a markedly different connectivity profile, primarily involving frontal executive-control regions and posterior occipital cortex, with no significant involvement of limbic or reward-related structures. Greater fussiness towards food was associated with increased connectivity between the right superior frontal gyrus (SFG) and associative cortex of the left occipital lobe (Rehman & Al Khalili, 2025). This may reflect a need for enhanced visual processing of food cues, related to fussy evaluation of food stimuli and their subsequent avoidance (Sader et al., 2025). Previous studies on young populations observed that selective, restrictive or avoidant eating patterns are often accompanied by increased reliance on sensory-perceptual features of food (e.g., appearance, texture) at the expense of reward-driven selection when making food choices (Rendall et al., 2022), and fronto-posterior activation that might sustain altered responsiveness towards food cues (Wang et al., 2016; Thomas et al., 2025). Fussy eating tendencies were also associated with increased connectivity between the right IFG and bilateral SFG/supplementary motor area (SMA), as well as with the left IFG and OFC. These regions are core hubs dedicated to inhibitory control and rule-based decision making (Friedman & Robbins, 2022), suggesting that food fussiness may be underpinned by the engagement of hyperresponsive regulatory mechanisms (Duck et al., 2025). Last, in our sample food fussiness was also positively correlated with both depression and anxiety aspects, coherently with previous findings on avoidant/restrictive eaters (Chammas et al., 2025). Collectively, these results suggest that food fussiness is accompanied by increased need for executive and evaluative control of the rewarding aspects of food, eventually resulting in a morbidly restrained approach to food selection.

The third aspect of food-related attitudes, undereating susceptibility, was associated with increased connectivity between the right hippocampus and the frontal operculum, anterior insula, and inferior frontal gyrus in the left hemisphere. This pattern implicates a network involved in interoceptive awareness, emotion-driven mnemonic retrieval, cognitive control and integration of bodily signals with higher-order decision making (Zhang et al., 2024). As addressed for overeating susceptibility, the hippocampus modulates eating and food choices based on former experiences with significant affective/rewarding salience (Kanoski & Grill, 2017; Stevenson & Francis, 2017). Interestingly, while overeating susceptibility was associated with left hippocampus-right insula connectivity, undereating susceptibility showed the opposite pattern. Though the hippocampi show partial functional lateralization (in the memory domain for instance (Robinson et al., 2015), how such lateralization affects human eating behavior is not yet known. Similarly, a functional lateralization may affect the capacity of this region to represent internal bodily states and primary physiological responses, including hunger and satiety (Wistehube et al., 2018; Huang et al., 2021). Greater connectivity between the right hippocampus and the insula may suggest an underlying enhanced integration between memory-based visual representations of prior eating experiences and physiological interoceptive signals. This could possibly bias one’s current food choices directing the individual toward restrictive or avoidant eating strategies, regardless of homeostatic needs or reward magnitude. Notably, altered hippocampal–insula connectivity has been reported in clinical populations with anorexia nervosa or subclinical restrictive eating, supporting the idea that such patterns may represent an early neural marker of vulnerability rather than a consequence of malnutrition (Favaro et al., 2012; Geisler et al., 2016; Frank et al., 2019).

Unlike overeating susceptibility, undereating susceptibility did *not* involve reward-related striatal regions, reinforcing the notion that restrictive eating tendencies may be driven more by interoceptive control mechanisms than the sensitivity to food and reward salience.

## Conclusion

The present findings demonstrate that definite appetitive traits in healthy adults are associated with discrete resting-state connectivity patterns which subsist in the absence of any overt clinical diagnosis of eating disorders or obesity. These results support multidimensional etiopathogenetic models of EDs and obesity, suggesting that vulnerability to these conditions may be embedded in early and stable brain network organizations, rather than emerging solely as consequence of overt symptomatology. Identifying such distinctive neural signatures in healthy populations, at any age, may help improve early detection strategies first, and then refining ad-hoc preventive interventions for both EDs and obesity.

## Supplementary Information

Below is the link to the electronic supplementary material.

Supplementary File 1 (DOCX 757 KB)

## Data Availability

The data that support the findings of this study are available from the corresponding author upon reasonable request.
