# Supplementary material for: Individual differences in eating attitudes are associated with distinct resting-state functional connectivity patterns in healthy adults
Source: Brain Imaging Behav. 2026 Nov 11;20(6):148. doi: 10.1007/s11682-026-01212-z (PMC13754994; doi:10.1007/s11682-026-01212-z)
Supplement: Supplementary file 1 [file 11682_2026_1212_MOESM1_ESM.docx]

Supplementary Materials

# 1SM. Hospital Anxiety and Depression Scale (HADS) and Fatigue Severity Scale (FSS)

Depressive and anxiety symptoms were evaluated using the HADS, a 14-items questionnaire that provides a quick, focused, yet comprehensive evaluation of mental health (Zigmond & Snaith, 1983). It relies on a 4-point Likert scale (0 = not at all, 3 = most of the time), with separate scores for anxiety and depression subscales (0–21 each). Cut-off scores are the following: 0–7 = Normal, 8–10 = Mild, 11–21 = Moderate to Severe.

FSS is a 9-items questionnaire meant to measure the severity of fatigue and its effects on daily life including interference with motivation, exercise, physical functioning, work and family life (Krupp et al., 1989). The FSS employs a 7-point Likert scale (1 = strongly disagree, 7 = strongly agree) for each of its nine items. A mean score of ≥ 4.0 is considered indicative of moderate to severe fatigue.

## 1SMa. HADS frequency graphs

## 1SMb. Non-parametric Spearman’s correlations between eating attitudes and psychological characteristics

Significant *positive* correlations have been found between, respectively, overeating susceptibility, depression scores (ρ = 0.503, p < .001), anxiety scores (ρ = 0.314, p = .030) and fatigue (ρ = 0.355, p = .013); between respectively food fussiness, depression scores (ρ = 0.376, p = .008) and anxiety scores (ρ = 0.367, p = .010). A significant *negative* correlation has been observed between food enjoyment and anxiety scores (ρ = -0.358, p = .013).

| ***Spearman's Correlations*** | | | |
| --- | --- | --- | --- |
| **Emotional Symptoms and Fatigue** | **Eating attitudes** | **Spearman's rho** | **p** |
| **Depression**  (HADS scores) | Overeating Susceptibility | **0.503***** | **< .001** |
|  | Food Enjoyment | -0.259 | .075 |
|  | Food Fussiness | **0.376**** | **.008** |
|  | Undereating Susceptibility | 0.203 | .166 |
| **Anxiety**  (HADS scores) | Overeating Susceptibility | **0.314*** | **.030** |
|  | Food Enjoyment | **-0.358*** | **.013** |
|  | Food Fussiness | **0.367*** | **.010** |
|  | Undereating Susceptibility | 0.183 | .212 |
| **Fatigue**  (Fatigue Severity Scale) | Overeating Susceptibility | **0.355*** | **.013** |
|  | Food Enjoyment | -0.031 | .837 |
|  | Food Fussiness | 0.259 | .075 |
|  | Undereating Susceptibility | 0.223 | .127 |
| *** p < .05, ** p < .01, *** p < .001** | | | |

# 2SM. Principal Component Analysis

## 2SMa. AEBQ Food Approach Dimension

| ***Components Loadings*** | | | |
| --- | --- | --- | --- |
| **AEBQ Subscales (Z scores)** | **Overeating Susceptibility**  **(RC1)** | **Food Enjoyment (RC2)** | **Uniqueness** |
| Food Responsiveness | 0.895 |  | 0.177 |
| Emotional Overeating | 0.866 |  | 0.210 |
| Hunger | 0.851 |  | 0.265 |
| Enjoyment of Food |  | 0.988 | 0.022 |
|  | | | |
|  | | | |

## 2SMb. AEBQ Food Avoidance Dimension

| ***Components Loadings*** | | | |
| --- | --- | --- | --- |
| **AEBQ Subscales (Z scores)** | **Food Fussiness (RC1)** | **Undereating Susceptibility (RC2)** | **Uniqueness** |
| Food Fussiness | 0.890 |  | 0.205 |
| Satiety Responsiveness | 0.771 |  | 0.200 |
| Slowness in Eating |  | 0.778 | 0.389 |
| Emotional Undereating |  | 0.711 | 0.402 |
|  | | | |
|  | | | |

# 3SM. AEBQ scores’ stratified descriptives

## 3SMa. AEBQ scores descriptives stratified by sex

| **AEBQ Variables** | | **Sex (n)** | **Median** | **Mean** | **SD** | **Range** | **Min** | **Max** |
| --- | --- | --- | --- | --- | --- | --- | --- | --- |
| **Dimensions** | Food Approach | F (28) | 2.950 | 2.888 | 0.477 | 2.113 | 2.063 | 4.175 |
|  |  | M (20) | 2.813 | 2.895 | 0.631 | 2.138 | 1.863 | 4.000 |
|  | Food Avoidance | F (28) | 2.419 | 2.458 | 0.435 | 1.725 | 1.438 | 3.163 |
|  |  | M (20) | 2.144 | 2.272 | 0.511 | 1.738 | 1.525 | 3.263 |
| **PCA-derived Eating Attitudes** | **Subscales** | **Sex** | **Median** | **Mean** | **SD** | **Range** | **Min** | **Max** |
| **Overeating Susceptibility** | Food Responsiveness | F (28) | 2.750 | 2.893 | 0.591 | 2.250 | 1.750 | 4.000 |
|  |  | M (20) | 2.750 | 2.873 | 0.739 | 2.500 | 2.000 | 4.500 |
|  | Emotional Overeating | F (28) | 2.000 | 2.171 | 0.820 | 3.000 | 1.000 | 4.000 |
|  |  | M (20) | 2.000 | 2.180 | 0.967 | 3.200 | 1.000 | 4.200 |
|  | Hunger | F (28) | 2.400 | 2.407 | 0.801 | 3.400 | 1.000 | 4.400 |
|  |  | M (20) | 2.300 | 2.428 | 0.889 | 2.800 | 1.000 | 3.800 |
| **Food Enjoyment** | Enjoyment of Food | F (28) | 4.300 | 4.079 | 0.532 | 2.000 | 2.600 | 4.600 |
|  |  | M (20) | 4.300 | 4.102 | 0.797 | 3.000 | 2.000 | 5.000 |
| **Food Fussiness** | Food Fussiness | F (28) | 1.800 | 1.750 | 0.803 | 4.000 | 1.000 | 5.000 |
|  |  | M (20) | 1.700 | 1.700 | 0.533 | 1.600 | 1.000 | 2.600 |
|  | Satiety Responsiveness | F (28) | 2.500 | 2.482 | 0.518 | 1.750 | 1.750 | 3.500 |
|  |  | M (20) | 2.125 | 2.295 | 0.779 | 2.750 | 1.250 | 4.000 |
| **Undereating Susceptibility** | Slowness Eating | F (28) | 2.375 | 2.564 | 0.920 | 3.500 | 1.000 | 4.500 |
|  |  | M (20) | 2.750 | 2.613 | 0.797 | 3.000 | 1.000 | 4.000 |
|  | Emotional Undereating | F (28) | 3.000 | 3.036 | 0.807 | 3.200 | 1.200 | 4.400 |
|  |  | M (20) | 2.600 | 2.480 | 0.946 | 3.000 | 1.000 | 4.000 |

## 3SMb. AEBQ scores descriptives stratified by educational level

| **AEBQ Variables** | | **Education (n)** | **Median** | **Mean** | **SD** | **Range** | **Min** | **Max** |
| --- | --- | --- | --- | --- | --- | --- | --- | --- |
| **Dimensions** | Food Approach | Primary (2) | 3.625 | 3.625 | 0.778 | 1.100 | 3.075 | 4.175 |
|  |  | Secondary (8) | 3.100 | 2.922 | 0.432 | 1.163 | 2.175 | 3.338 |
|  |  | Higher (38) | 2.669 | 2.846 | 0.535 | 2.138 | 1.863 | 4.000 |
|  | Food Avoidance | Primary (2) | 2.606 | 2.606 | 0.168 | 0.238 | 2.488 | 2.725 |
|  |  | Secondary (8) | 2.238 | 2.353 | 0.621 | 1.713 | 1.525 | 3.238 |
|  |  | Higher (38) | 2.394 | 2.374 | 0.454 | 1.825 | 1.438 | 3.263 |
| **PCA-derived Eating Attitudes** | **Subscales** | **Education** | **Median** | **Mean** | **SD** | **Range** | **Min** | **Max** |
| **Overeating Susceptibility** | Food Responsiveness | Primary (2) | 3.500 | 3.500 | 0.707 | 1.000 | 3.000 | 4.000 |
|  |  | Secondary (8) | 3.250 | 3.088 | 0.715 | 1.750 | 2.000 | 3.750 |
|  |  | Higher (38) | 2.750 | 2.809 | 0.624 | 2.750 | 1.750 | 4.500 |
|  | Emotional Overeating | Primary (2) | 3.600 | 3.600 | 0.566 | 0.800 | 3.200 | 4.000 |
|  |  | Secondary (8) | 2.800 | 2.575 | 0.671 | 2.000 | 1.200 | 3.200 |
|  |  | Higher (38) | 2.000 | 2.016 | 0.840 | 3.200 | 1.000 | 4.200 |
|  | Hunger | Primary (2) | 3.100 | 3.100 | 1.838 | 2.600 | 1.800 | 4.400 |
|  |  | Secondary (8) | 2.400 | 2.425 | 0.705 | 2.400 | 1.200 | 3.600 |
|  |  | Higher (38) | 2.300 | 2.378 | 0.813 | 2.800 | 1.000 | 3.800 |
| **Food Enjoyment** | Enjoyment of Food | Primary (2) | 4.300 | 4.300 | 0.000 | 0.000 | 4.300 | 4.300 |
|  |  | Secondary (8) | 4.000 | 3.600 | 0.882 | 2.300 | 2.000 | 4.300 |
|  |  | Higher (38) | 4.300 | 4.180 | 0.569 | 2.400 | 2.600 | 5.000 |
| **Food Fussiness** | Food Fussiness | Primary (2) | 1.900 | 1.900 | 0.141 | 0.200 | 1.800 | 2.000 |
|  |  | Secondary (8) | 1.600 | 1.750 | 0.657 | 1.600 | 1.000 | 2.600 |
|  |  | Higher (38) | 1.700 | 1.716 | 0.730 | 4.000 | 1.000 | 5.000 |
|  | Satiety Responsiveness | Primary (2) | 2.500 | 2.500 | 0.000 | 0.000 | 2.500 | 2.500 |
|  |  | Secondary (8) | 2.450 | 2.519 | 0.882 | 2.750 | 1.250 | 4.000 |
|  |  | Higher (38) | 2.250 | 2.375 | 0.606 | 2.250 | 1.250 | 3.500 |
| **Undereating Susceptibility** | Slowness Eating | Primary (2) | 2.625 | 2.625 | 0.530 | 0.750 | 2.250 | 3.000 |
|  |  | Secondary (8) | 2.375 | 2.419 | 0.709 | 2.500 | 1.250 | 3.750 |
|  |  | Higher (38) | 2.750 | 2.617 | 0.914 | 3.500 | 1.000 | 4.500 |
|  | Emotional Undereating | Primary (2) | 3.400 | 3.400 | 0.283 | 0.400 | 3.200 | 3.600 |
|  |  | Secondary (8) | 2.800 | 2.725 | 0.907 | 2.800 | 1.200 | 4.000 |
|  |  | Higher (38) | 2.800 | 2.789 | 0.924 | 3.400 | 1.000 | 4.400 |
